# Novel anti-inflammatory peptide alleviates liver ischemia-reperfusion injury

**DOI:** 10.7555/JBR.38.20240020

**Published:** 2024-05-29

**Authors:** Xuejun Xu, Kaineng Sun, Hao Chang, Chunxiang Shen, Xiangdong Li, Yangyue Ni, Yuxiao Zhu, Huiquan Wang, Ruiyan Xiong, Jon Rob Padde, Zhipeng Xu, Lin Chen, Lu Chen, Min Hou, Liyong Pu, Minjun Ji

**Affiliations:** 1 Department of Pathogen Biology, National Vaccine Innovation Platform, Jiangsu Province Engineering Research Center of Antibody Drug, School of Basic Medical Sciences, Nanjing Medical University, Nanjing, Jiangsu 211166, China; 2 Hepatobiliary Center, Jiangsu Province Hospital and Nanjing Medical University First Affiliated Hospital, Nanjing, Jiangsu 210029, China

**Keywords:** schistosome-derived peptide, liver ischemia-reperfusion injury, macrophage, toll-like receptor-4

## Abstract

Ischemia-reperfusion injury (IRI) remains an unavoidable challenge in liver surgery, with macrophages playing a critical role in its pathogenesis. However, the mechanisms by which macrophages regulate the pathogenesis of IRI are not well understood. Through a target-guided screening approach, we identified a small 3 kDa peptide (SjDX5-271) from various schistosome egg-derived peptides that induced M2 macrophage polarization. SjDX5-271 treatment protected mice against liver IRI by promoting M2 macrophage polarization, and this protective effect was abrogated when the macrophages were depleted. Transcriptomic sequencing showed that the TLR signaling pathway was significantly inhibited in macrophages from the SjDX5-271 treatment group. We further identified that SjDX5-271 promoted M2 macrophage polarization by inhibiting the TLR4/MyD88/NF-κB signaling pathway and alleviated hepatic inflammation in liver IRI. Collectively, SjDX5-271 exhibited some promising therapeutic effects in IRI and represented a novel therapeutic approach, potentially applicable to other immune-related diseases. The current study demonstrates the potential of new biologics from the parasite, enhances our understanding of host-parasite interplay, and provides a blueprint for future therapies for immune-related diseases.

## Introduction

Ischemia-reperfusion injury (IRI) is a common complication of liver surgery, caused by cell damage resulting from the disrupted blood flow, which is further aggravated by reperfusion^[1]^. The organ damage caused by IRI may lead to acute or chronic rejection. Liver IRI is a complex process including multiple mechanisms, such as oxidative stress, apoptosis, and inflammatory response^[2]^. The resulting local inflammation is further aggravated by the recruitment of leukocytes, especially macrophages, which mediates liver IRI^[3]^. However, the exact mechanism of immune regulation is not completely clear.

Inflammatory responses of innate immune cells are the primary cause of liver IRI. During liver ischemia, immune cells release reactive oxygen species and pro-inflammatory cytokines, such as tumor necrosis factor-α (TNF-α) and interleukin (IL)-6, which are key contributors to this stage. Additionally, immunogenic cellular contents released during apoptosis and necrosis, such as the damage-associated molecular patterns, exacerbate the inflammatory cascade. During liver reperfusion, immune cells are recruited to the injury site *via* blood flow, further exacerbating the inflammation^[4]^. As the most abundant hepatic innate immune cells, Kupffer cells (KCs) constitute approximately 20% of the non-parenchymal cells and play a key role in initiating and amplifying inflammatory responses during liver IRI^[5]^. KCs are resident macrophages in the liver characterized by their functional diversity and plasticity. According to their phenotype and function, macrophages are usually classified into M1 macrophages with a pro-inflammatory phenotype and M2 macrophages with an anti-inflammatory function. M2-type macrophages are further categorized into M2a (IL-4/IL-13 stimulated), M2b (immune complexes and Toll-like-receptor or IL-1R agonists stimulated), and M2c (IL-10 stimulated)^[6]^. The state of macrophages is not static but transforms between M1 and M2. During liver IRI, M1 macrophages produce pro-inflammatory cytokines, such as TNF-α, IL-6, and IL-1β, to promote inflammation, while M2 macrophages release anti-inflammatory cytokines, such as IL-10, transforming growth factor-β, and arginase-1, to repair tissues and reduce inflammation^[7]^. Therefore, understanding the immunomodulatory mechanisms of macrophages is crucial for developing novel therapeutic strategies to mitigate IRI.

During co-evolution with humans, parasites have developed complex immune evasion mechanisms, including the secretion of biologically active substances that suppress host immune responses and protect the parasites^[8]^. The schistosome egg antigen (SEA) is a complex mixture of biologically active molecules produced by *Schistosoma* *japonicum* eggs and exhibits potent immunoregulatory effects, including the regulation of M2 macrophages^[9]^. Therefore, the anti-inflammatory properties and active components of SEA have been extensively studied. In previous studies, many short peptides were isolated from egg extracts of the helminth *S.* *japonicum* using gel filtration chromatography and reversed-phase high-performance liquid chromatography^[10]^.

In the current study, we identified a small 3 kDa peptide (SjDX5-271) among various schistosome egg-derived peptides to explore its role and mechanisms in liver IRI.

## Materials and methods

### Microarray data

The GSE151648 dataset, with the sample type of Homo sapiens, was obtained from the Gene Expression Omnibus (GEO) (https://www.ncbi.nlm.nih.gov/gds/). This dataset contained liver RNA-seq data from 17 IRI^−^ patients and 23 IRI^+^ patients before and after transplantation, respectively. All analyses were performed using R studio 4.2.1. Differential expression analyses were performed using the DESeq2 R package. Genes with |log_2_(fold change)| > 0.585 and *P*-value < 0.05 were characterized as differentially expressed genes (DEGs) between the IRI^−^ and IRI^+^ groups after transplantation. The filtered DEGs were used to create volcano maps. The Kyoto Encyclopedia of Genes and Genomes (KEGG) and Gene Ontology (GO) enrichment analyses of the DEGs were performed using the cluster Profiler R package. Those with a *P*-value < 0.05 were considered significantly enriched. The single-sample gene set enrichment analysis (ssGSEA) of the R package gsva was used to analyze cell expression differences in the immune microenvironment between the IRI^−^ and IRI^+^ groups.

### Animals

Male C57BL/6 mice (aged 6 to 8 weeks) and the toll-like receptor 4 (TLR4)-deficient (*Tlr4*^−/−^) mice were housed in a pathogen-free environment at the Animal Core Facility of Nanjing Medical University. The mice were maintained under controlled conditions (22 ℃, 55% humidity, and a 12-h diurnal cycle). The animal care and use protocols were approved by the Institutional Animal Care and Use Committee (IACUC) of Nanjing Medical University (Approval No. 2112057). All experiments were performed in strict accordance with the Regulations for the Administration of Affairs Concerning Experimental Animals.

### Peptides

The SjDX5-271 was previously prepared from *S.* *japonicum* eggs in our laboratory and identified by mass spectrum analysis^[11]^, comprising 30 amino acids (95% purity; sequence: YKNLGGQQQSGSSQGQFPSGQMQQQQRPQQ). The control peptide sequence was generated by randomly arranging the SjDX5-271 sequence (95% purity; sequence: GQQPQGSNKYNQQPSQSGFLQSGQQMRQQG). All peptides were synthesized by Sangon Biotech (Nanjing, Jiangsu, China).

### Cell culture and treatment

The RAW264.7 cell line was cultured in DMEM supplemented with 10% fetal bovine serum (FBS), penicillin (100 U/mL), and streptomycin (100 mg/mL). Cells were cultured in an incubator at 37 ℃ in an atmosphere containing 5% CO_2_. Cell viability was determined using the CCK-8 (Beyotime, Shanghai, China) assay. RAW264.7 cells were seeded in a 96-well plate at a density of 5000 cells/well and then stimulated with different concentrations of SjDX5-271 (100, 50, 25, 12.5, 6.25, 3.13, 1.56, 0.78, and 0.39 μg/mL) for 24 h. After treatment, the CCK-8 solution was added to the medium, followed by incubation at 37 ℃ for 2 h. Absorbance was measured at 450 nm using a microplate reader (Bio-Rad, Hercules, CA, USA).

A human monocyte leukemia cell line (THP-1) was cultured at a density of 5 × 10^5^ cells/mL in an RPMI 1640 medium supplemented with 10% FBS and 1% penicillin/streptomycin solution at 37 ℃ in an incubator containing 5% CO_2_. THP-1 monocytes were cultured in 12-well plates and treated with 100 nmol/L phorbol 12-myristate 13-acetate for 24 h for transformation into adherent macrophages.

Bone marrow-derived macrophages (BMDMs) were prepared as previously described^[12]^. Bone marrow cells were flushed from the femur and tibia of mice and grown in DMEM (Gibco, Waltham, MA, China) supplemented with 10% heat-inactivated FBS, 1% antibiotics, and granulocyte-macrophage colony-stimulating factor (20 ng/mL of M-CSF; PeproTech, Cranbury, NJ, USA). The medium was changed every two days. After seven days, the cells were mature and ready for use. RAW264.7, THP-1, and BMDMs were all treated with lipopolysaccharide (LPS; 0.5 μg/mL) with or without SjDX5-271 (10 μg/mL) for 24 h.

### Liver IRI model

A liver IRI mouse model was established as previously described^[13]^. The mice were anesthetized *via* inhalation of isoflurane and underwent a midline laparotomy to expose the liver tissues. An atraumatic clip was then used to cut off the arterial and portal blood supply to the severed liver lobes. Seventy percent of the liver tissues were ischemic for 90 min, after which the forceps were removed to initiate reperfusion. The mice were placed on thermostatic pads to maintain their body temperature. The sham-operated mice underwent the same procedure but without blood vessel occlusion. The mice were euthanized 6 h after perfusion to obtain the liver and serum samples. In the liver IRI mouse model, liver injury peaks in terms of liver inflammation and necrotic area at 6 h after reperfusion. Therefore, to evaluate the ability of SjDX5-271 to inhibit inflammation, we chose to perform reperfusion for 6 h to detect relevant indicators. SjDX5-271 (3 mg/kg), control peptide (3 mg/kg), or vehicle saline was administered intravenously at 24 h before ischemia.

Mice in the macrophage depletion group received 200 μL of blank liposomes and clodronate liposomes (CLD; Yesen, Shanghai, China) intravenously at 48 h before liver ischemia surgery. SjDX5-271 was injected intravenously at 24 h before the liver ischemia surgery.

### Measurement of the serum transaminase levels

The levels of serum alanine transaminase (ALT) and aspartate transaminase (AST) were measured by an automatic biochemistry analyzer (Beckman Coulter, Pasadena, CA, USA).

### Histopathology assay and immunofluorescence staining

Harvested liver tissues were fixed in 4% formalin and embedded in paraffin blocks. Sections with a thickness of 4 μm were prepared and stained with hematoxylin and eosin (H&E). Liver IRI was evaluated by a pathologist in a blinded manner employing the Suzuki criteria and scored on a scale ranging from 0 to 4 points. According to the degrees of liver congestion, vacuole-like degeneration, and cell necrosis, scores were given as asymptomatic (0 points), very mild (< 10% of the area, 1 point), mild (11%–30% of the area, 2 points), moderate (31%–60% of the area, 3 points), and severe (> 60% of the area, 4 points)^[14]^.

Frozen liver samples (4 μm) were fixed with acetone and methanol (1∶1) for 130 min and permeabilized by Triton X-100 for 30 min. Subsequently, non-specific binding sites were blocked with 0.2% BSA for 1 h, followed by incubation with the primary antibody at 4 ℃ overnight. Primary antibodies used in the current study included: rabbit anti-F4/80 (1∶200; Cat. #28463-1-AP, Proteintech, Wuhan, China), mouse anti-CD86 (1∶1000; Cat. #ab269587, Abcam, Cambridge, England), and anti-CD206 (1∶1000; Cat. #ab252921, Abcam). The slides were washed and incubated with anti-mouse or anti-rabbit secondary antibodies at room temperature for 2 h, and the nuclei were counterstained for 3 min for cell localization. The images were acquired using a fluorescence microscope^[15]^.

The TUNEL (Beyotime) staining was used to assess apoptosis in the liver, and procedures were performed according to the manufacturer's instructions.

### Real-time reverse transcription-PCR (qRT-PCR)

Total RNA was extracted from liver tissues or cells using TRIzol reagent (Vazyme, Nanjing, China) and reversely transcribed into cDNA using a cDNA reverse transcription kit (Vazyme). qPCR was performed using the SYBR Green Master Mix kit (Vazyme), and detection was performed using the LightCycler® 96 real-time PCR system (Roche, Basel, Switzerland). The amplification program was as follows: 1 cycle at 95 ℃ for 30 s, followed by 40 cycles at 95 ℃ for 10 s, 60 ℃ for 30 s, and 72 ℃ for 30 s. Relative expression of related genes was calculated using the 2^−ΔΔct^ method, and the expression was normalized to that of *GAPDH*. The qPCR primer sequences are listed in ***Supplementary Table 1*** (available online).

### Western blotting analysis

Proteins were extracted from liver IRI tissues and cultured with BMDM using RIPA Lysis Buffer (Beyotime) for Western blotting. Protein concentrations were determined using the BCA Protein Assay Kit (Beyotime). Equal amounts of proteins (50 μg) were separated by 10% sodium dodecyl sulfate-polyacrylamide gel electrophoresis (SDS-PAGE) and transferred to polyvinylidene difluoride membranes (Bio-Rad, Hercules, CA, USA). Membranes were blocked in 5% nonfat milk and then incubated overnight at 4 ℃ with the following primary antibodies: rabbit anti-BAX antibody (1∶2000; Cat. #50599-2-lg, Proteintech), rabbit anti-BCL2 antibody (1∶2000; Cat. #4223T, Cell Signaling Technology, Danvers, MA, USA), mouse anti-TLR4 antibody (1∶2000; Cat. #66350-1-1g, Proteintech), rabbit anti-MyD88 antibody (1∶2000; Cat. #23230-1-AP, Proteintech), rabbit anti-p65 antibody (1∶2000; Cat. #80979-1-RR, Proteintech), rabbit anti-p-p65 antibody (1∶2000; Cat. #82335-1-RR, Proteintech), and mouse β-actin monoclonal antibody (1∶5000; Cat. #66009-1-1g, Proteintech). β-Actin was used as a control. On the next day, membranes were incubated with peroxidase-conjugated goat anti-rabbit or goat anti-mouse IgG for 1 h at room temperature and subjected to substrate development.

### Enzyme-linked immunosorbent assay (ELISA)

Cell culture supernatants and mouse sera were collected for cytokine expression analysis. Cytokine secretion (IL-6 and IL-10) was measured by ELISA according to the manufacturer's protocols (Thermo Fisher Scientific, Waltham, MA, USA).

### Biotin pull-down assay

BMDMs were lysed in an IP lysate supplemented with 0.5 mmol/L PMSF (Sigma, St. Louis, MO, USA). A total of 150 μg of biotin was added to cell lysates containing Streptavidin Magnetic Beads (NEB, Suzhou, China) and rotated at 4 ℃ overnight, then the magnetic beads were removed from cell lysates to eliminate non-specific binding. The biotinylated peptide and magnetic beads were reincubated with pretreated cell lysates at 4 ℃ overnight. The beads were washed thoroughly with ice-cold PBS, and the complexes were eluted. Protein separation and detection were performed by SDS-PAGE and Western blotting, respectively.

### Library construction for RNA-seq and sequencing procedures

Total RNA (*n* = 2) was extracted from BMDMs treated with LPS or LPS + SjDX5-271 using an RNeasy Mini Kit (Qiagen, Dusseldorf, Germany). Paired-end libraries were synthesized by using the TruSeq® RNA Sample Preparation Kit (Illumina, San Diego, CA, USA) following the TruSeq® RNA Sample Preparation Guide. Briefly, poly (A)-containing mRNA was purified using poly (T) oligo-attached magnetic beads. Purified libraries were quantified by Qubit 2.0 Fluorometer (Thermo Fisher Scientific) and validated by Agilent 2100 Bioanalyzer (Agilent Technologies, Santa Clara, CA, USA) to determine the insert size and calculate the molar concentration. After dilution to 10 pmol/L, clusters were generated using cBot with the library and sequenced on an Illumina HiSeq X-ten (Illumina). Library construction and sequencing were performed at Shanghai Biotechnology Corporation (Shanghai, China). Fold change was estimated from the fragments per kilobase per million mapped reads (FPKM) for each sample. The screening criteria for differentially expressed genes were false discovery rate ≤ 0.05 and fold-change ≥ 1.5.

### Statistical analysis

All experiments were performed in triplicates and repeated independently. All statistical analyses were performed using GraphPad 7.0. Data were shown as mean ± standard error of the mean. Normally distributed data were compared using the Student's *t*-test. A *P*-value < 0.05 was considered statistically significant.

## Results

### Macrophages were involved in human liver ischemia-reperfusion injury

**Figure 1:**
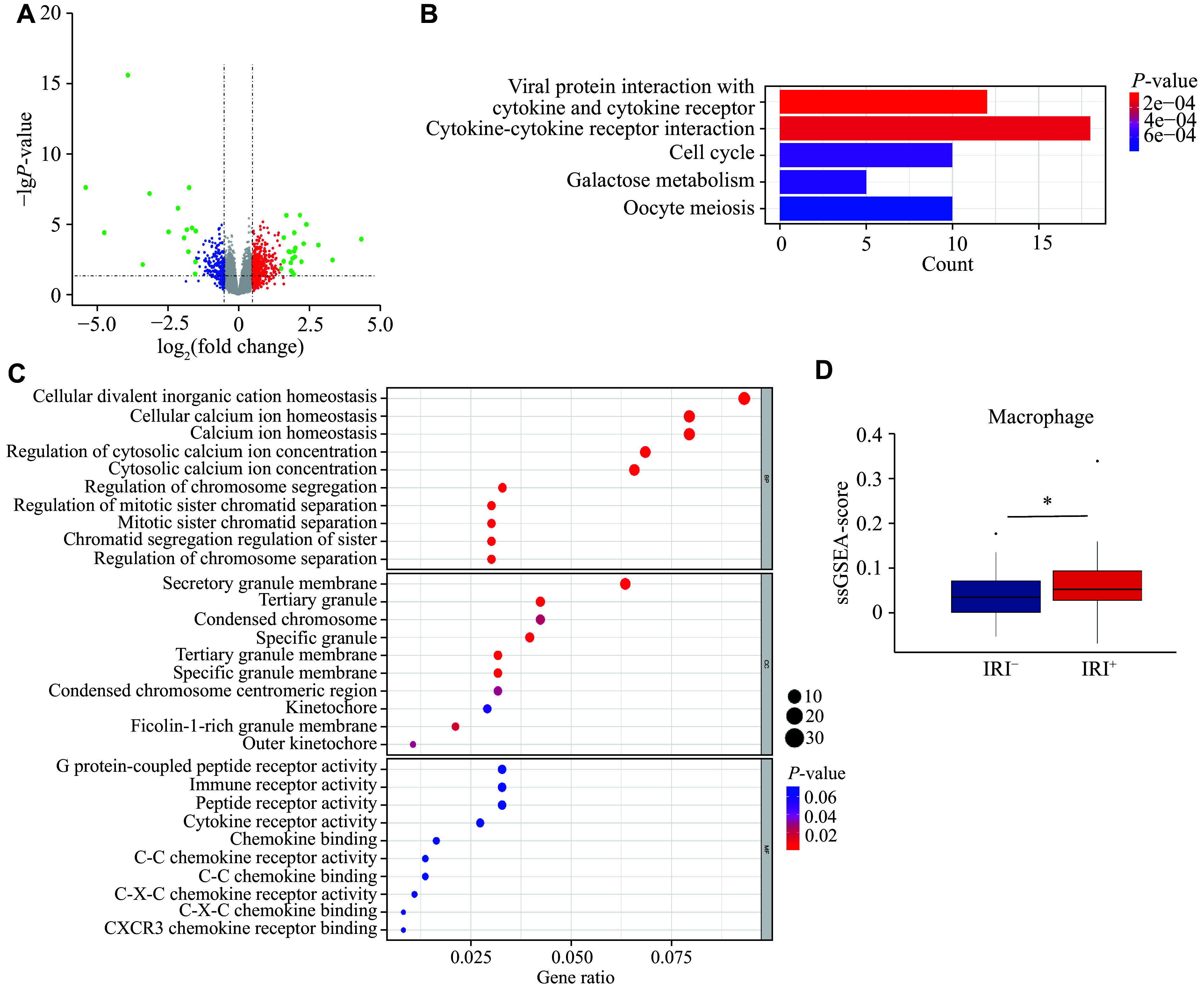
Macrophages were involved in the regulation of ischemia-reperfusion injury (IRI). A: A volcano map of the differentially expressed genes (DEGs) between the IRI^−^ and IRI^+^ groups. Red represents up-regulated genes, and blue represents down-regulated genes. B: The KEGG pathway analysis for the most significantly enriched signaling pathways of the aforementioned DEGs. C: The Gene Ontology analysis of the aforementioned DEGs. D: The single-sample gene set enrichment analysis (ssGSEA) on immune infiltration in the liver tissues from both IRI^−^ and IRI^+^ groups. ^*^P < 0.05.

We downloaded the GSE151648 dataset from the GEO database. This dataset includes liver samples from 17 IRI^−^ and 23 IRI^+^ patients before and after liver transplantation, respectively, and contains transcriptome data related to human liver IRI. We analyzed DEGs in the IRI^−^ and IRI^+^ groups after transplantation, and screened 440 DEGs from the dataset, including 296 upregulated and 144 downregulated genes (***Fig. 1A***). Enrichment analysis showed that the DEGs were significantly enriched in the cytokine-cytokine receptor interaction signaling pathway (***Fig. 1B***). GO functional analysis further revealed that the DEGs were significantly enriched in the molecular functions of cytokine and immune receptor activities (***Fig. 1C***). These results indicated that immune cells played an important role in liver ischemic perfusion. A study has shown that the activation of pro-inflammatory macrophages plays an important role in this process^[16]^. Therefore, we analyzed the infiltration of macrophages in the two groups and found that the number of phagocytes increased significantly in the IRI^+^ group compared with that in the IRI^−^ group (***Fig. 1D***).

### Treatment with SjDX5-271 reduced the LPS-induced M1 polarization *in vitro*

**Figure 2:**
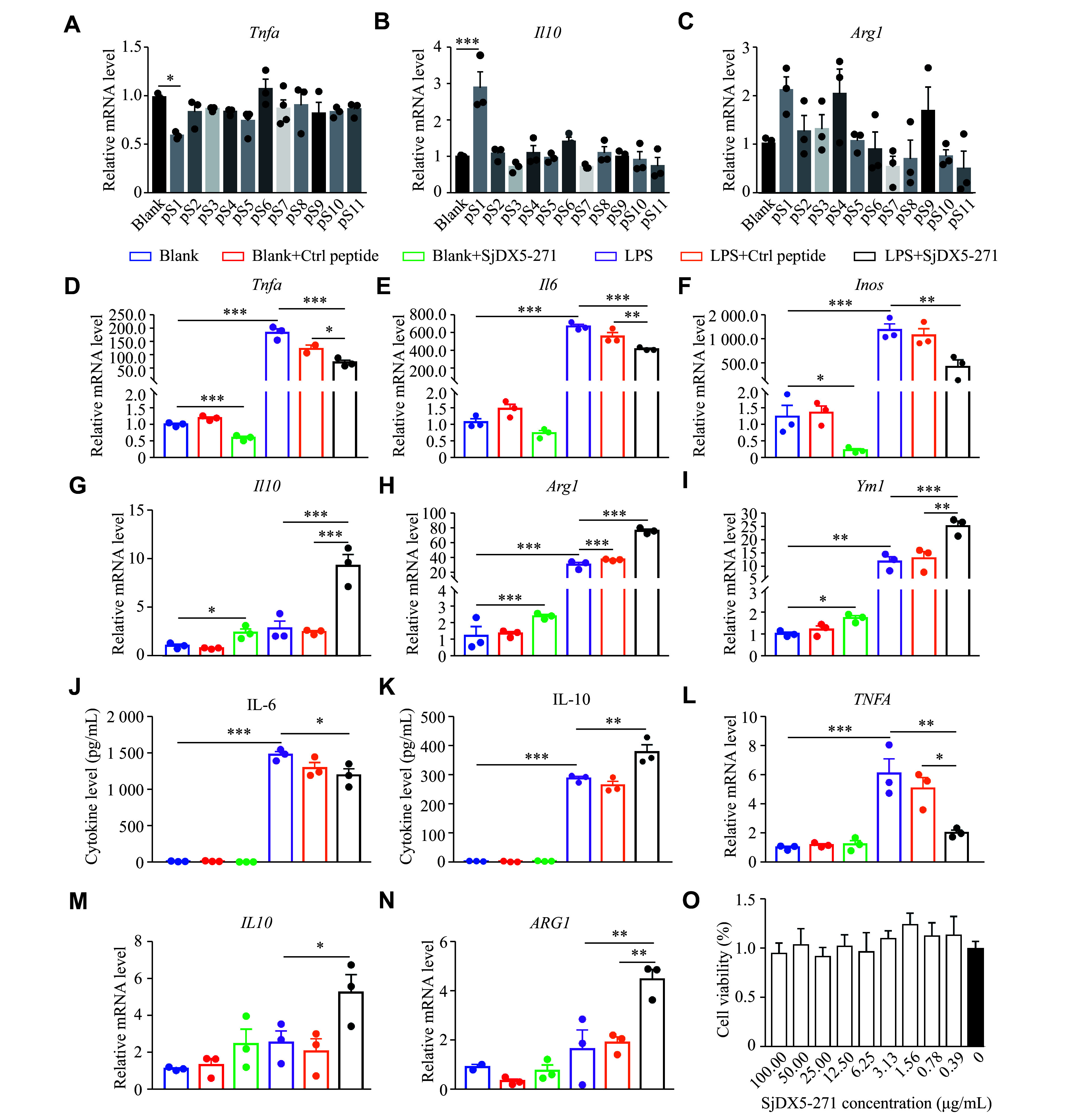
SjDX5-271 treatment reduced the lipopolysaccharide (LPS)-induced M1 polarization *in vitro*. A–C: RAW264.7 cells were stimulated with 11 peptides (10 μg/mL) isolated from schistosome eggs for 24 h (*n* = 3), and the mRNA expression levels of *Tnfα* (A), *Il10* (B), and *Arg1* (C) were quantitatively detected by qRT-PCR. D–K: RAW264.7 cells were incubated with control (Ctrl) peptide (10 μg/mL) or SjDX5-271 (10 μg/mL) with or without LPS (0.5 μg/mL) for 24 h, and the mRNA expression levels of *Tnfα* (D), *Il6* (E), *Inos* (F), *Il10* (G), *Arg1* (H) and *Ym1* (I) were detected by qRT-PCR (*n* = 3). The cell supernatant was collected and the cytokine levels of IL-6 (J) and IL-10 (K) were detected by ELISA (*n* = 3). L–N: THP-1 cells were stimulated with control peptide (10 μg/mL) or SjDX5-271 (10 μg/mL) with or without LPS (0.5 μg/mL) for 24 h, and the mRNA expression levels of *TNFA* (L), *IL10* (M), and *ARG1* (N) were quantitatively detected by qRT-PCR (*n* = 3). O: RAW264.7 cells were stimulated with different concentrations of SjDX5-271 for 24 h, and cell viability was detected by the CCK-8 assay (*n* = 3). Data are shown as mean ± standard error of the mean. Normally distributed data were compared using the Student's *t*-test. Significant differences are represented as ^*^P < 0.05, ^**^P < 0.01, and ^***^P < 0.001.

We prepared 11 peptides from *S.* *japonicum* eggs and evaluated their anti-inflammatory properties in macrophages^[11]^. We found that pS1 significantly induced M2 polarization in RAW264.7 cells (***Fig. 2A***–***2C***). Based on its source, pS1 was named SjDX5-271. To investigate the regulatory functions of SjDX5-271, we cultured murine BMDMs *in vitro*, stimulated them with SjDX5-271, and examined the mRNA levels of various cytokines in these BMDMs. The results showed that the LPS stimulation significantly increased the mRNA levels of M1-related markers (*i.e.*, *Tnfa*, *Il6*, and *Inos*). However, this enhancement was inhibited by the SjDX5-271 treatment (***Fig. 2D***–***2F***), which stimulated the transcription of M2-related markers (*i.e.*, *Il10*, *Arg1*, and *Ym1*) (***Fig. 2G***–***2I***). We also collected culture supernatants from the SjDX5-271-stimulated BMDMs and detected the secretion levels of IL-6 and IL-10. The results showed that the IL-6 secretion levels were significantly reduced, whereas the IL-10 secretion levels were significantly increased in the SjDX5-271-stimulated group than in the LPS treatment group, and there was also no significant difference between the LPS treatment and the LPS + control peptide group (***Fig. 2J*** and ***2K***). Similarly, SjDX5-271 also induced M2 polarization in THP-1 cells (***Fig. 2L***–***2N***). Finally, we assessed the cytotoxicity of SjDX5-271 against RAW264.7 cells using the CCK-8 assay, and found that the viability of RAW264.7 cells was not affected by SjDX5-271 treatment at various concentrations (***Fig. 2O***). These results indicated that SjDX5-271 induced the polarization of M2 macrophages and inhibited the LPS-induced M1 macrophage polarization *in vitro*.

### SjDX5-271 pretreatment ameliorated IRI in mice

**Figure 3:**
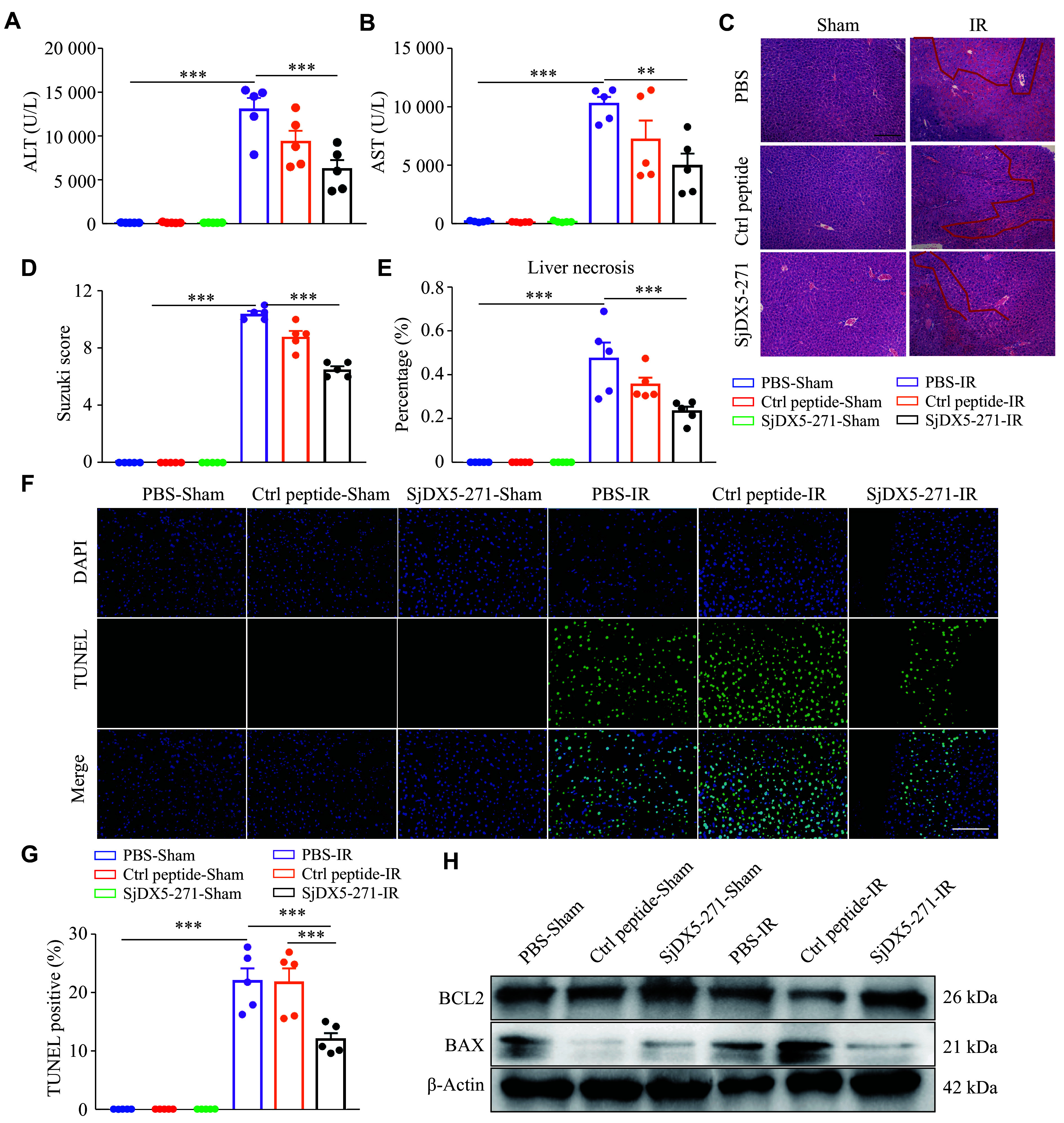
SjDX5-271 pretreatment ameliorated hepatic ischemia-reperfusion injury (IRI) in mice. The mice were injected with the specified concentration of control (Ctrl) peptide (3 mg/kg) and SjDX5-271 (3 mg/kg) through the tail vein for 24 h and then underwent liver ischemia reperfusion surgery. After ischemia for 90 min, the mice were sutured layer by layer on the abdomen, and the mice were euthanized after perfusion for 6 h. The sham operation group received the same treatment except for no ischemic treatment. A–B: The concentrations of ALT (A) and AST (B) in the serum were detected by automatic biochemistry analyzer (*n* = 5). C–E: Paraffin-embedded liver sections were stained with hematoxylin and eosin after surgical treatment (*n* = 5), using the Suzuki score and liver necrosis to quantitatively assess the level of liver damage. The red line shows the ischemic degeneration of the liver (C). F–G: Representative liver sections showing TUNEL staining to assess the rate of apoptosis after IRI (*n* = 5). Scale bar, 200 μm. H: The levels of apoptosis-related proteins BAX and BCL-2 in the liver tissues of mice were detected by Western blotting after perfusion for 6 h (*n* = 3). Data are shown as mean ± standard error of the mean. Normally distributed data were compared using the Student's *t*-test. Significant differences are represented as ^**^P < 0.01 and ^***^P < 0.001.

To investigate the effect of SjDX5-271 on liver injury, we established the liver IRI model in mice. Pretreatment with SjDX5-271 in the IRI group significantly decreased the serum ALT and AST levels, markers commonly used to assess the degree of liver damage and to reflect liver function, compared with the IRI group (***Fig. 3A*** and ***3B***). The morphology of mouse livers was analyzed by the H&E staining. Mice with IRI showed extensive necrosis with massive hepatic inflammatory cell infiltration and sinusoidal congestion. SjDX5-271 pretreatment alleviated these symptoms (***Fig. 3C***). In addition, the liver injury scores, assessed using the Suzuki scoring method, were significantly higher in the IRI group than in the SjDX5-271-pretreated IRI group (***Fig. 3D***). The necrotic area was significantly smaller in the SjDX5-271-pretreated IRI group than in the IRI group (***Fig. 3E***). Furthermore, a reduced percentage of TUNEL-positive cells was observed in the SjDX5-271-pretreated IRI group compared with the IRI and control peptide-pretreated IRI group (***Fig. 3F*** and ***3G***). A decreased expression level of pro-apoptotic BAX, along with an increased expression level of anti-apoptotic BCL2, was observed in the SjDX5-271-pretreated IRI group, compared with the IRI group (***Fig. 3H***; ***Supplementary Fig. 1***, available online). These results indicated that SjDX5-271 pretreatment prevented liver IRI in mice.

### SjDX5-271 suppressed inflammation during liver IRI by promoting hepatic M2 macrophage polarization

**Figure 4:**
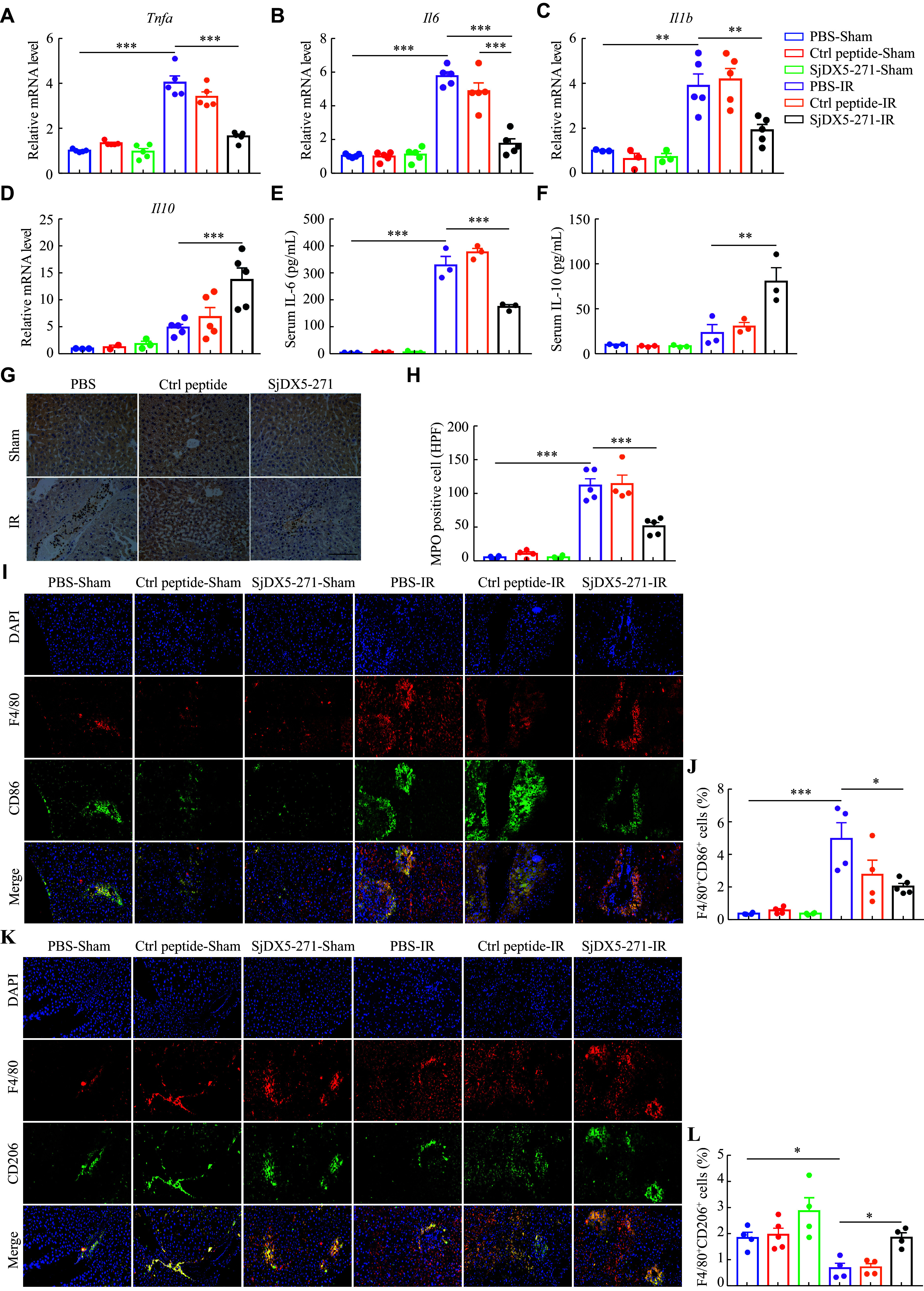
SjDX5-271 attenuated inflammatory responses during liver ischemia-reperfusion (IRI). After injecting control (Ctrl) peptide (3 mg/kg) or SjDX5-271 (3 mg/kg) into the tail vein, the mice were sacrificed after liver IRI surgery, and their liver tissues and serum were collected. A–D: The mRNA expression of *Tnfα* (A), *Il6* (B), *Il1b* (C), and *Il10* (D) in the liver tissues was detected by qRT-PCR (*n* = 5). E–F: The concentrations of IL-6 (E) and IL10 (F) in the serum were detected by ELISA (*n* = 3). G–H: The expression of MPO in the liver tissues was determined by immunohistochemical staining (G), and the number of MPO-positive cells was counted (H) (*n* = 5). Scale bar, 200 μm. I–L: Immunofluorescence of F4/80^+^ (red), CD86^+^ (green), and CD206^+^ (green) in the liver tissues was detected, and the immunofluorescence staining positive cell area statistics were performed (*n* = 4). Scale bar, 200 μm. Data are shown as mean ± standard error of the mean. Normally distributed data were compared using the Student's *t*-test. Significant differences are represented as ^*^P < 0.05, ^**^P < 0.01, and ^***^P < 0.001.

A large amount of pro-inflammatory cytokines is produced during IRI. Therefore, we investigated the effect of SjDX5-271 pretreatment on the inflammatory response after liver IRI in mice. We found that the mRNA levels of *Tnfa,* *Il6,* and *Il1b* were significantly decreased, while those of anti-inflammatory cytokine (*Il10*) were significantly increased in the SjDX5-271 pretreatment group after liver IRI, compared with the IRI group (***Fig. 4A***–***4D***). We further examined the expression levels of IL-6 and IL-10 in the serum, and found that SjDX5-271 pretreatment significantly reduced IL-6 but increased IL-10 secretion (***Fig. 4E*** and ***4F***). In addition, we examined the expression of MPO to assess neutrophil cell infiltration in liver sections. Immunohistochemical analysis showed that MPO expression was significantly inhibited after pretreatment with SjDX5-271 (***Fig. 4G*** and ***4H***). Because macrophages play an important role in IRI, we subsequently examined the expression of macrophages in the liver tissues. The immunofluorescence results showed an increased infiltration of hepatic macrophages after IRI, which was manifested by an increase in the number of F4/80-positive cells. The proportion of F4/80^+^CD86^+^ M1 macrophages was significantly decreased, but that of F4/80^+^CD206^+^ M2 macrophages was significantly increased in the SjDX5-271-pretreated IRI group than in the IRI group (***Fig. 4I***–***4L***). These results indicated that SjDX5-271 attenuated liver IRI by regulating hepatic macrophage polarization.

### SjDX5-271 exerted a protective effect *via* macrophages during IRI

**Figure 5:**
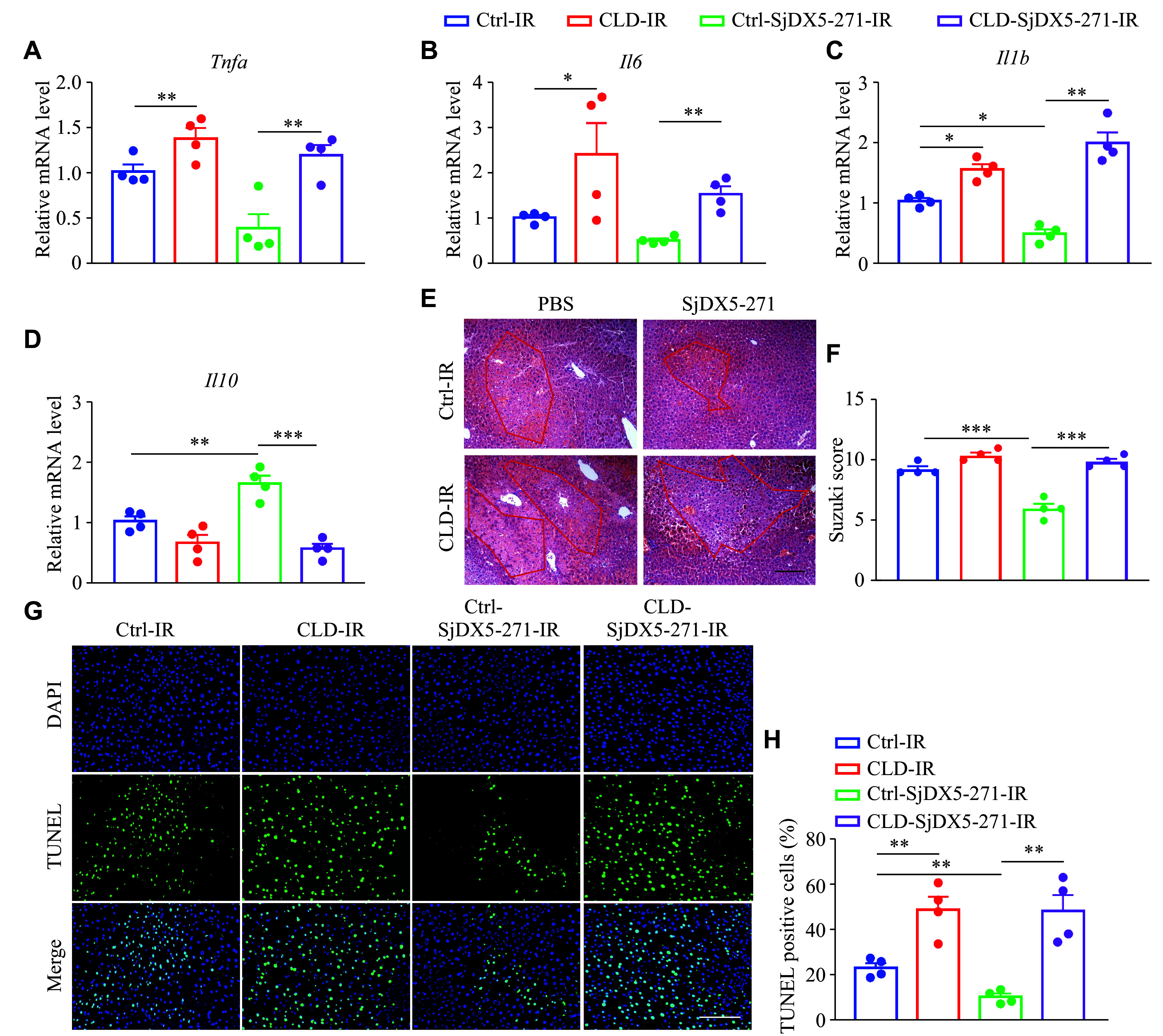
SjDX5-271 exerted anti-inflammatory effects through macrophages during liver ischemia-reperfusion injury (IRI). After intraperitoneally injecting 200 μL of bland liposomes (Ctrl) or clodronate liposomes (CLD) for 24 h, followed by tail vein injection of SjDX5-271 (3 mg/kg) for 24 h, the mice underwent liver surgery and were sacrificed after reperfusion for 6 h, and then the liver tissues were isolated. A–D: The mRNA expression levels of *Tnfα* (A), *Il6* (B), *Il1b* (C), and *Il10* (D) in the liver tissues were detected by qRT-PCR (*n* = 4). E–F: Paraffin-embedded liver sections were stained with H&E (*n* = 4), The red line is the area of hepatic ischemic degeneration, and the liver damage was calculated with the Suzuki score. G–H: Representative liver sections show TUNEL staining to assess the rate of apoptosis after IR (*n* = 4). Scale bar, 200 μm. Data are shown as mean ± standard error of the mean. Normally distributed data were compared using the Student's *t*-test. Significant differences are represented as ^*^P < 0.05, ^**^P < 0.01, and ^***^P < 0.001.

To further test whether the protective effect of SjDX5-271 on liver IRI was macrophage-dependent, macrophages were depleted by intravenous injection of CLD at 48 h before liver IRI. Compared with the blank liposome-treated mice, the CLD-treated mice showed an increased liver injury, as evidenced by the elevated expression levels of pro-inflammatory cytokines (*i.e.*, *Tnfa*, *Il6*, and *Il1b*) and the decreased expression levels of anti-inflammatory cytokine *Il10* (***Fig. 5A***–***5D***; ***Supplementary Fig.2***, available online). Moreover, H&E and TUNEL staining demonstrated that liver necrosis in mice was aggravated after CLD treatment (***Fig. 5E***–***5H***). These results indicated that the protective effect of SjDX5-271 against liver IRI was abrogated by macrophage depletion, and that SjDX5-271 exerted the protective effects in a macrophage-dependent manner.

### SjDX5-271 regulated macrophage polarization *via* the TLR4/MyD88/NF-κB signaling pathway

**Figure 6:**
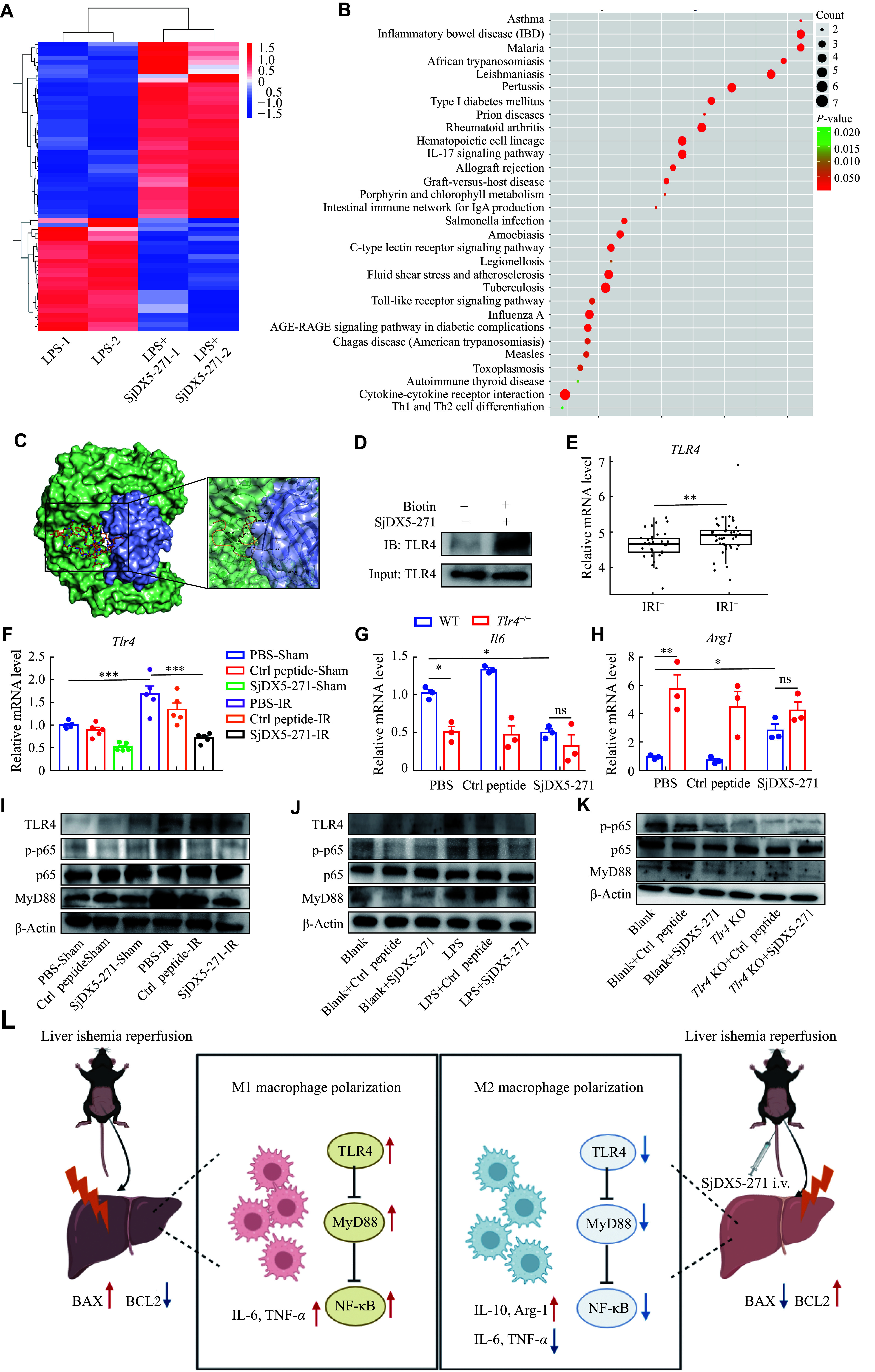
SjDX5-271 regulated the TLR4/MyD88/NF-κB expression to control macrophage polarization. A–B: After the wild-type (WT) mouse BMDMs were differentiated and matured *in vitro*, RNA was extracted for transcriptome sequencing after 24 h stimulation with lipopolysaccharide (LPS; 0.5 μg/mL) and SjDX5-271 (10 μg/mL) (*n* = 2). The heatmap (A) shows transcriptionally altered genes induced by the SjDX5-271 treatment in BMDMs upon LPS stimulation. The KEGG analysis (B) shows related pathways altered by SjDX5-271. C: Molecular docking was used to predict the binding site of TLR4 protein to SjDX5-271. The green and purple modules are TLR4 complex, and the peptide segment is SjDX5-271. D: The biotin pull-down assay between biotin-SjDX5-271 and TLR4 in BMDMs detected by Western blotting (WB). E: Differential expression of *Tlr4* was analyzed in the GSE151648 dataset between the IRI^−^ and IRI^+^ groups after liver transplantation. F: The *Tlr4* mRNA expression in the liver tissues was detected by qRT-PCR (*n* = 5). G–H: After differentiation and maturation of BMDMs from WT or *Tlr4*^−/−^ *in vitro*, the mRNA expression levels of *Il6* (G) and *Arg1* (H) were detected by qRT-PCR after 24 h stimulation with control (Ctrl) peptide (10 μg/mL) or SjDX5-271 (10 μg/mL) (*n* = 3). I: The protein expression levels of TLR4, p65, phosphorylated p65 (p-p65), and MyD88 in the liver tissues were detected by WB (*n* = 3). J: BMDMs were treated with SjDX5-271 (10 μg/mL) with or without lipopolysaccharide (LPS; 0.5 μg/mL) for 24 h. The expression levels of TLR4, p65, p-p65, and MyD88 were analyzed by WB (*n* = 3). K: After differentiation and maturation of BMDMs from WT or *Tlr4*^−/−^ mice and stimulation with Ctrl peptide or SjDX5-271 for 24 h, WB was used to detect the expression levels of p65, p-p65 and MyD88 proteins (*n* = 3). L: The schematic illustration indicates that SjDX5-271 promotes the M2 macrophage polarization and inhibits the TLR4/MyD88/NF-κB pathway. Data are shown as mean ± standard error of the mean. Normally distributed data were compared using the Student's *t*-test. Significant differences are represented as ^*^P < 0.05, ^**^P < 0.01, and ^***^P < 0.001.

To identify the factors mediating the acquisition of the anti-inflammatory phenotype in macrophages stimulated by SjDX5-271, we performed a transcriptome analysis between the LPS-stimulated and the LPS + SjDX5-271-stimulated BMDMs using a high-throughput RNA-Seq. As shown, 39 DEGs were upregulated and 25 were downregulated in BMDMs from the LPS + SjDX5-271 group, compared with those from the LPS group (***Fig. 6A***). The KEGG pathway analysis showed that the TLR signaling pathway was significantly inhibited in macrophages derived from the LPS + SjDX5-271 group, suggesting that SjDX5-271 may have a potential modulatory effect on the TLR-mediated signaling pathway (***Fig. 6B***).

Using molecular docking (http://huanglab.phys.hust.edu.cn/hpepdock/), SjDX5-271 was predicted to bind to TLR4 (***Fig. 6C***). To further verify the interaction between SjDX5-271 and TLR4, we performed biotin pull-down assays, which showed that endogenously expressed TLR4 was enriched in biotin-labeled SjDX5-271 (***Fig. 6D***). The analysis of RNA-seq results in human liver tissues of the IRI^−^ and IRI^+^ groups showed that the *TLR4* expression levels were significantly increased after liver transplantation (***Fig. 6E***). We then examined the mRNA levels of *Tlr4* in the mouse liver tissues, and found that the expression levels of *Tlr4* were increased in the IRI group, which was significantly reversed after SjDX5-271 pretreatment (***Fig. 6F***). To further determine whether SjDX5-271 exerts its protective effect through a TLR4-dependent mechanism, we isolated BMDMs from both WT and *Tlr4*^−/−^ mice, and examined the mRNA levels of *Il6* and *Arg1*. The results showed that SjDX5-271 did not induce a decrease in *Il6* expression or an increase in *Arg1* expression, when *Tlr4* was knocked out (***Fig. 6G*** and ***6H***). The Western blotting results also showed that the TLR4 levels were significantly increased after liver IRI, but that SjDX5-271 pretreatment significantly reduced the TLR4 expression (***Fig. 6I***; ***Supplementary Fig. 3A***, available online). These results indicated that the anti-inflammatory properties of SjDX5-271 might be mediated by TLR4.

Myeloid differentiation factor 88 (MyD88) and NF-κB are downstream effectors of the TLR4 signaling pathway. Hence, we examined the protein levels of MyD88 and NF-κB in mouse liver tissues. The results showed that SjDX5-271 significantly downregulated the protein levels of MyD88 and NF-κB phosphorylated p65 (***Fig. 6I***; ***Supplementary Fig. 3A***). In BMDMs stimulated with LPS with or without SjDX5-271, the protein levels of TLR4, MyD88, and p-p65 were significantly increased after LPS stimulation, whereas their expression levels were significantly decreased after SjDX5-271 treatment (***Fig. 6J***; ***Supplementary Fig. 3B***, available online). Both *in vivo* and *in vitro* results in mice demonstrated that SjDX5-271 exhibited a significant anti-inflammatory effect. To investigate whether the anti-inflammatory function of SjDX5-271 depends on TLR4, we extracted BMDMs from *Tlr4*^−/−^ mice, and found that the inhibitory effect of SjDX5-271 on MyD88 and p-p65 was attenuated in the absence of TLR4 (***Fig. 6K***; ***Supplementary Fig. 3C*** and ***3D***, available online). Collectively, these results indicated that the anti-inflammatory effects of SjDX5-271 might be TLR4-dependent.

## Discussion

IRI may lead to severe liver damage and pose a significant clinical challenge during liver surgery. Different therapeutic strategies have been developed to target various mechanisms of liver IRI^[17–18]^. In the early stages of liver IRI, KCs polarize toward M1 macrophages, releasing pro-inflammatory cytokines that aggravate liver injury. In the current study, we found that SjDX5-271 significantly suppressed the pro-inflammatory response and improved liver IRI in model mice. This protective effect was achieved by promoting M2 macrophage polarization and inhibiting the TLR4/MyD88/NF-κB pathway. SjDX5-271 is a promising peptide for clinical applications and demonstrates potential as a novel therapeutic agent for the improvement of liver injury after liver surgery and transplantation (***Fig. 6L***).

Studies have shown that the inflammatory responses mediated by liver macrophages are key to the development of IRI^[19]^. After liver transplantation and reperfusion, macrophages are activated to resist the production of endogenous injury-associated molecular patterns and pathogen-associated molecular patterns to repair damaged tissues^[20]^. However, the overactivation of macrophages may result in pro-inflammatory immune responses and the increased liver damage. Simultaneously, circulating monocytes are recruited to the liver, resulting in significant macrophage infiltration following liver reperfusion. Although macrophages increase liver damage during the reperfusion, some studies have shown that the depletion of macrophages in mice may lead to the increased liver damage and even death^[21]^. SjDX5-271 alleviated liver inflammation by significantly inducing the differentiation of macrophages into M2 macrophages. Consistent with previous findings, we observed exacerbated hepatic IRI after macrophage depletion^[22]^, and the protective effects of SjDX5-271 on the liver were circumvented by macrophage depletion. These results demonstrate that macrophages play an important role in IRI regulation. We have demonstrated, for the first time, that SjDX5-271 alleviates IRI by regulating macrophages.

Schistosomal components effectively modulate inflammatory responses and autoimmune diseases. These components typically induce an innate immune response, followed by an adaptive immune response. SEA is a complex mixture of many important biologically active molecules produced by *S.* *japonicum* eggs and exhibits potent immunoregulatory effects, including suppressing dextran sulfate sodium-induced colitis and improving ovalbumin-induced asthma symptoms^[23–24]^. It has also been shown that SEA-treated macrophages exhibit M2 polarization phenotype *in vitro*^[25]^. Although SEA plays an important role in regulating immunity, its constituent parts are complex and challenging to access. Therefore, screening for a single anti-inflammatory component is crucial for future translational applications of SEA. Peptides, because of their characteristics, such as low molecular weight, high biological safety, and easy absorption, have been widely used in research to solve basic biological problems and have demonstrated potential in preclinical and clinical settings^[26–27]^. At present, hirudin 66 peptide is used as an anticoagulant in clinical treatment of various thrombotic diseases^[28–29]^. Based on this, we screened for anti-inflammatory peptides in SEA and found that SjDX5-271 had the strongest ability to induce the polarization of M2 macrophages. SjDX5-271 induced the production of anti-inflammatory cytokines, such as *Il10* and *Arg1*. As an anti-inflammatory cytokine, IL-10 plays an important role in protection against IRI. Therefore, we hypothesize that SjDX5-271 induces the polarization of M2 macrophages along with the secretion of anti-inflammatory cytokines, such as IL-10, by inhibiting the activation of the TLR4/MyD88/NF-κB signaling pathway. In addition, we demonstrated that SjDX5-271 alleviated the inflammatory infiltration of neutrophils. This may be an important reason for the relief from inflammatory injury. Our study provides novel insights into the parasite-associated proteins involved in the treatment of immune-related diseases as sources of next-generation biologics.

Toll-like receptors are pattern recognition receptors that play key roles in the response to different structurally conserved components of pathogens^[30]^. Among them, TLR4 plays a crucial role in ischemic injury in the brain, heart, kidney, and liver^[31–32]^. Through bioinformatic predictions and experimental validation, we found that SjDX5-271 might bind to TLR4 to reduce the pro-inflammatory response. Furthermore, when TLR4 was eliminated in mice, the anti-inflammatory activity of SjDX5-271 disappeared, demonstrating that TLR4 is an important target of SjDX5-271. The TLR4 signaling pathway triggers the activation of downstream MyD88 and NF-κB signaling pathways and further mediates the release of numerous pro-inflammatory cytokines^[33]^. The current study showed that the expression of TLR4, MyD88, and p-p65 significantly decreased after SjDX5-271 treatment. Thus, SjDX5-271 regulated M2-type macrophage polarization by inhibiting the TLR4/MyD88/NF-κB signaling pathway, thereby alleviating hepatic inflammation in liver IRI. When TLR4 was inhibited, the polarization of M2-type macrophages increased, which was consistent with our results^[34]^. However, several questions remain unanswered. The liver contains various cell types and is the largest internal organ in the body. In addition to macrophages, other cells, including hepatocytes^[35]^, are also involved in the development of liver IRI. Significant research efforts are still required to elucidate the anti-inflammatory mechanism of SjDX5-271 in the context of acute liver injury.

In conclusion, the current study elucidated the protective effect of SjDX5-271 in promoting macrophage M2 polarization by inhibiting the TLR4/MyD88/NF-κB signaling pathway. SjDX5-271 exhibits potential therapeutic effects against IRI. We report a novel parasite-isolated biologic that advances our understanding of the host-parasite interplay and may provide a blueprint for the treatment of immune-related diseases and drug development in the future.

## SUPPLEMENTARY DATA

Supplementary data to this article can be found online.
