# Supplementary material for: Novel anti-inflammatory peptide alleviates liver ischemia-reperfusion injury
Source: J Biomed Res. 2024 May 29;39(1):61–75. doi: 10.7555/JBR.38.20240020 (PMC11873596; doi:10.7555/JBR.38.20240020)
Supplement: Supplementary file 1 — Supplementary data to this article can be found online. [file jbr-39-1-61-S1.pdf]

Available online at www.jbr-pub.org.cn

Open Access at PubMed Central

JBR

Journal of Biomedical Research, 2025 39(1): 1–3

Supplementary Data

Novel anti-inflammatory peptide alleviates liver ischemia-reperfusion injury

Xuejun Xu[1], Kaineng Sun[1], Hao Chang[1], Chunxiang Shen[1], Xiangdong Li[2], Yangyue Ni[1], Yuxiao Zhu[1], Huiquan Wang[1], Ruiyan Xiong[1], Jon Rob Padde[1], Zhipeng Xu[1], Lin Chen[1], Lu Chen[1], Min Hou[1], Liyong Pu[2], Minjun Ji[1,✉]

[1]Department of Pathogen Biology, National Vaccine Innovation Platform, Jiangsu Province Engineering Research Center of Antibody Drug, School of Basic Medical Sciences, Nanjing Medical University, Nanjing, Jiangsu 211166, China;
[2]Hepatobiliary Center, Jiangsu Province Hospital and Nanjing Medical University First Affiliated Hospital, Nanjing, Jiangsu 210029, China.

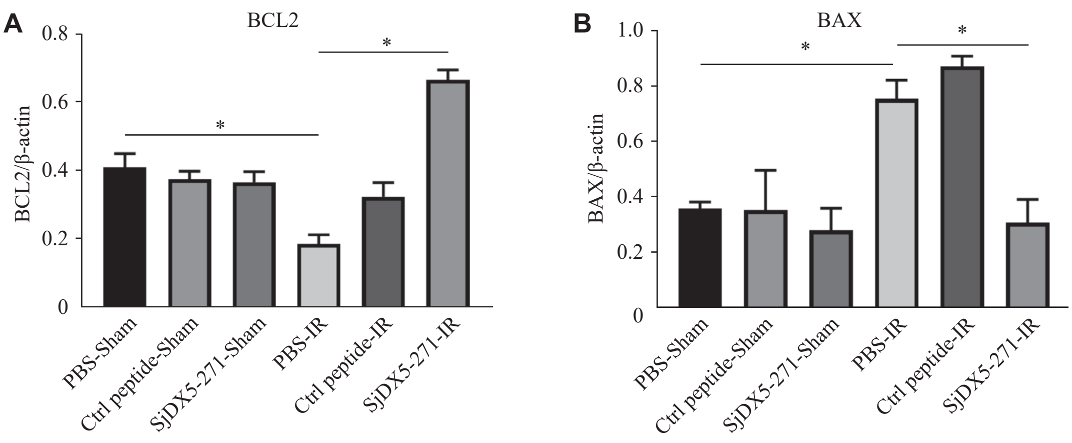

Supplementary Fig. 1 **The band quantitative data of BCL2 and BAX by Western blotting.** The band quantitative data of BCL2 (A) and BAX (B) were analyzed in mouse liver tissues. Data are shown as mean ± standard error of the mean. Normally distributed data were compared using the Student's *t*-test. Significant differences are represented as $^{*}P < 0.05$.

✉Corresponding author: Minjun Ji, Department of Pathogen Biology, National Vaccine Innovation Platform, Jiangsu Province Engineering Research Center of Antibody Drug, School of Basic Medical Sciences, Nanjing Medical University, 101 Longmian Road, Jiangning District, Nanjing, Jiangsu 211166, China. E-mail: jiminjun@njmu.edu.cn.

Received: 22 January 2024; Revised: 11 April 2024; Accepted: 30 April 2024; Published online: 29 May 2024

CLC number: R657.3, Document code: A

The authors reported no conflict of interests.

This is an open access article under the Creative Commons Attribution (CC BY 4.0) license, which permits others to distribute, remix, adapt and build upon this work, for commercial use, provided the original work is properly cited.

© 2025 by Journal of Biomedical Research.

https://doi.org/10.7555/JBR.38.20240020

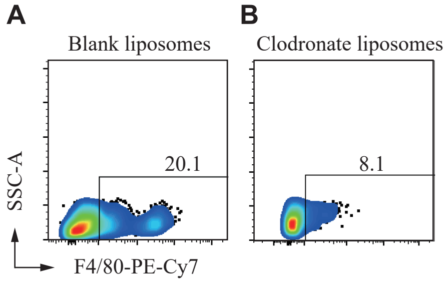

Supplementary Fig. 2 **The elimination effect on the groups of blank liposomes and clodronate liposomes.** The proportion of macrophages eliminated by blank liposomes (A) and clodronate liposomes (B) was detected by flow cytometry in mouse liver tissues.

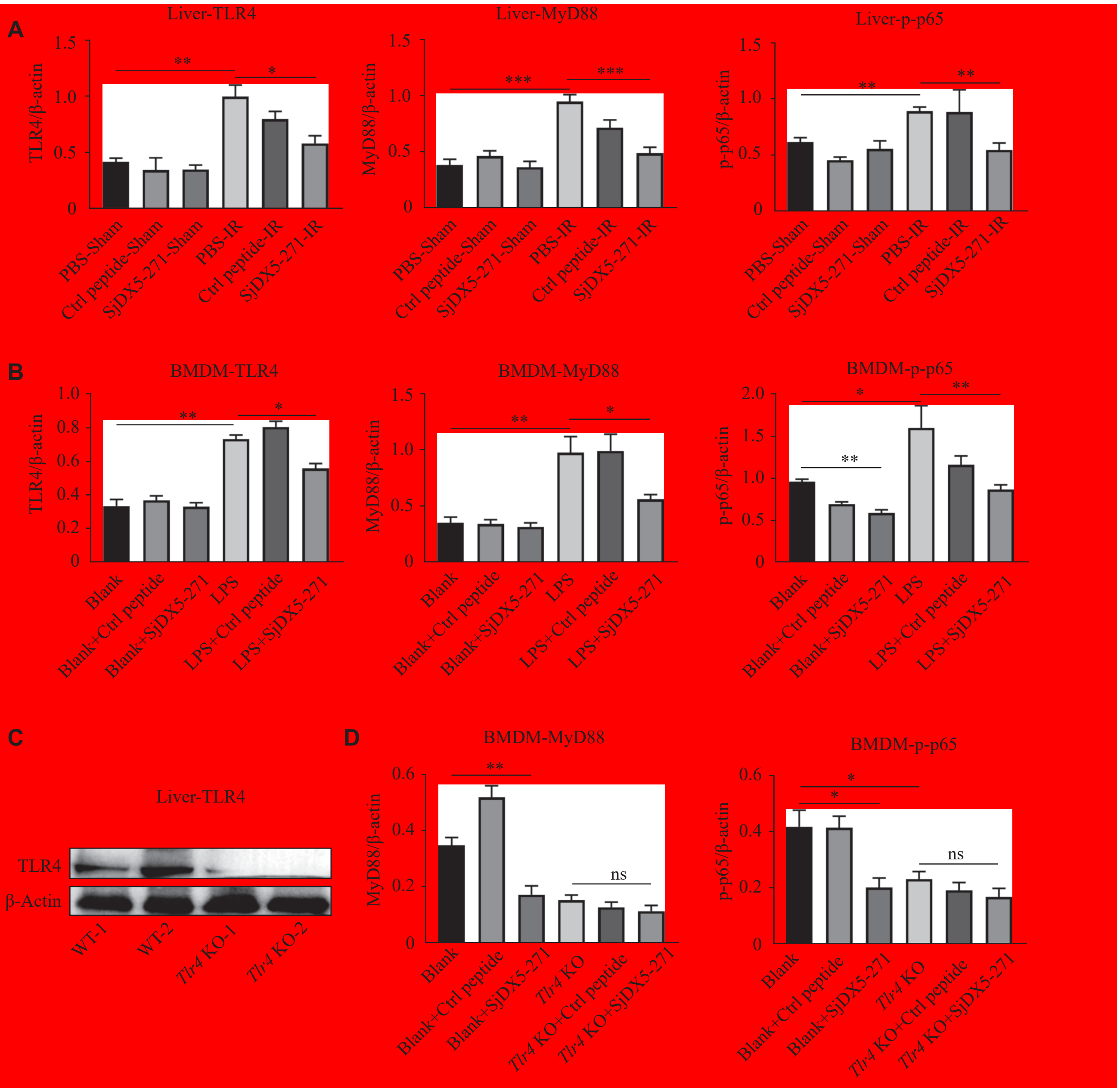

Supplementary Fig. 3 **The band quantitative data by Western blotting.** A: The band quantitative data of TLR4, MyD88, and p-p65 were analyzed in mouse liver. B: The band quantitative data of TLR4, MyD88, and p-p65 were analyzed in BMDMs cells in the presence or absence of LPS. C: The expression of TLR4 was determined by Western blotting in *TLR4*$^{-/-}$ mice liver. D: The band quantitative data of MyD88 and p-p65 were analyzed in *TLR4*$^{-/-}$ mice BMDMs in the absence or presence of SjDX5-271. Data are shown as mean ± standard error of the mean. Normally distributed data were compared using the Student's *t*-test. Significant differences are represented as $^{*}P < 0.05$, $^{**}P < 0.01$, and $^{***}P < 0.001$.

Supplementary Table 1 **Primers used for quantitative reverse transcription PCR**

Target genes	Forward primers (5′-3′)	Reverse primers (5′-3′)
Il1b	GAAATGCCACCTTTTGACAGTG	TGGATGCTCTCATCAGGACAG
Tnfa	CATCTTCTCAAAATTCGAGTGACAA	TGGGAGTAGACAAGGTACAACCC
Il6	GAGGATACCACTCCCAACAGACC	AAGTGCATCATCGTTGTTCATACA
Inos	GCCACCAACAATGGCAACA	CGTACCGGATGAGCTGTGAATT
Il10	ACTTTAAGGGTTACTTGGGTTGC	ATTTTCACAGGGGAGAAATCG
Arg1	CAGAAGAATGGAAGAGTCAG	CAGATATGCAGGGAGTCACC
Ym1	TCACAGGTCTGGCAATTCTTCTG	TTTGTCCTTAGGAGGGCTTCCTC
Gapdh	GGTGAAGGTCGGTGTGAACG	ACCATGTAGTTGAGGTCAATGAAG
TNFA	GCCTGTACCTCATCTACTC	CCTTGGTCTGGTAGGAGA
INOS	GGAGGTGCTAGAGGAGTT	AGGAGCTGATGGAGTAGAA
IL10	GAAAGGCATCTACAAAGC	GTTTCGTATCTTCATTGTCA
ARG1	GTGTGATGTGAAGGATTATGG	TTCTTCCGTTCTTCTTGACT
GAPDH	TATGACAACAGCCTCAAGAT	AGTCCTTCCACGATACCA
